# Reduction of Phosphorylated Synapsin I (Ser-553) Leads to Spatial Memory Impairment by Attenuating GABA Release after Microwave Exposure in Wistar Rats

**DOI:** 10.1371/journal.pone.0095503

**Published:** 2014-04-17

**Authors:** Simo Qiao, Ruiyun Peng, Haitao Yan, Yabing Gao, Changzhen Wang, Shuiming Wang, Yong Zou, Xinping Xu, Li Zhao, Ji Dong, Zhentao Su, Xinxin Feng, Lifeng Wang, Xiangjun Hu

**Affiliations:** 1 Department of Experimental Pathology, Beijing Institute of Radiation Medicine, Beijing, People’s Republic of China; 2 Department of Radiation Protection and Health Physics, Beijing Institute of Radiation Medicine, Beijing, People’s Republic of China; 3 Department of Biochemical Pharmacology, Beijing Institute of Pharmacology and Toxicology, Beijing, People’s Republic of China; 4 Endocrine and Cardiovascular Center, Cardiovascular Institute and Fuwai Hospital of Chinese Academy of Medical Sciences, Beijing, People’s Republic of China; Aston University, United Kingdom

## Abstract

**Background:**

Abnormal release of neurotransmitters after microwave exposure can cause learning and memory deficits. This study investigated the mechanism of this effect by exploring the potential role of phosphorylated synapsin I (p-Syn I).

**Methods:**

Wistar rats, rat hippocampal synaptosomes, and differentiated (neuronal) PC12 cells were exposed to microwave radiation for 5 min at a mean power density of 30 mW/cm^2^. Sham group rats, synaptosomes, and cells were otherwise identically treated and acted as controls for all of the following post-exposure analyses. Spatial learning and memory in rats was assessed using the Morris Water Maze (MWM) navigation task. The protein expression and presynaptic distribution of p-Syn I and neurotransmitter transporters were examined via western blotting and immunoelectron microscopy, respectively. Levels amino acid neurotransmitter release from rat hippocampal synaptosomes and PC12 cells were measured using high performance liquid chromatograph (HPLC) at 6 hours after exposure, with or without synapsin I silencing via shRNA transfection.

**Results:**

In the rat experiments, there was a decrease in spatial memory performance after microwave exposure. The expression of p-Syn I (ser-553) was decreased at 3 days post-exposure and elevated at later time points. Vesicular GABA transporter (VGAT) was significantly elevated after exposure. The GABA release from synaptosomes was attenuated and p-Syn I (ser-553) and VGAT were both enriched in small clear synaptic vesicles, which abnormally assembled in the presynaptic terminal after exposure. In the PC12 cell experiments, the expression of p-Syn I (ser-553) and GABA release were both attenuated at 6 hours after exposure. Both microwave exposure and p-Syn I silencing reduced GABA release and maximal reduction was found for the combination of the two, indicating a synergetic effect.

**Conclusion:**

p-Syn I (ser-553) was found to play a key role in the impaired GABA release and cognitive dysfunction that was induced by microwave exposure.

## Introduction

Microwaves have become extremely important for many industries, particularly in communication and medical fields. With increasing understanding of the latent health hazards of microwave exposure, it is becoming clear that effective methods for protection and treatment of those working in the presence of microwaves are urgently needed. It has been reported that frequent occupational microwave exposure can result in diverse and complex diseases [1], [2], especially behavioral disturbances due to dysfunction of the central nervous system [3]. Although this system is one of the most sensitive targets of microwave exposure, the detailed mechanisms underlying microwave-induced effects on behavior and cognitive ability remain unclear.

Amino acid neurotransmitters in the mammalian brain play critical roles in the neural processes relating to cognitive function. Under normal conditions, they are packed into synaptic vesicles by vesicular neurotransmitter transporters and released into the synaptic cleft via exocytosis. Each transporter subtype acts as a specific marker of neurons containing that particular neurotransmitter (or structurally related neurotransmitter), e.g., vesicular GABA transporter (VGAT) and vesicular glutamate transporter 1 (VGLUT1) found in GABAergic and glutamatergic neurons, respectively [4]. Cognitive and memory ability are dependent on a coordinated program of neurotransmitter release from nerve terminals by presynaptic exocytosis, a process that is regulated by synaptic vesicular proteins [5].

Synapsin I, is one of the most important of these, and it is known to regulate the efficiency of neurotransmitter release. Synapsin I is predominantly involved in regulation of synaptic vesicle trafficking at pre-docking stages by maintaining the balance between readily-releasable and reserve synaptic vesicles at the presynaptic membrane [6]–[8]. Synapsin I itself is regulated via its phosphorylation status; phosphorylated synapsin I (p-Syn I) plays an important role in the final post-docking steps of exocytosis including vesicular priming and fusion [9], [10].

Numerous experiments have indicated that hippocampus-dependent spatial learning and memory deficits occurring after microwave exposure are associated with the impairment of hippocampal neurotransmission [11]–[14], but whether and how p-Syn I is involved requires elucidation. Therefore, the aim of this study was to investigate the potential role of p-Syn I in microwave-induced impairment of synaptic transmission.

## Materials and Methods

### Ethics Statement

All protocols were approved by the Institutional Animal Care and Use Committee of Beijing Institute of Radiation Medicine.

### Animals

Male Wistar rats (200±20 g; *N* = 90) were obtained from the Laboratory Animal Center (Beijing, China) and maintained at 22±2°C with a 12 hour light-dark cycle (lights on at 7 a.m.). Food and water were freely available, and all efforts were made to minimize suffering.

### Cell Culture

Cells of the PC12 line, originally isolated from rat pheochromocytoma [15], were cultured in Dulbecco’s modified eagle’s medium (DMEM; Invitrogen, Carlsbad, CA, USA), supplemented with 10% (v/v) fetal bovine serum, 5% (v/v) horse serum, 5% 100 U/ml penicillin, and 0.1 mg/ml streptomycin. All cells were cultured in a sterile incubator maintained at 37°C with 5% CO_2_. Neuronal differentiation of PC12 cells was induced by supplementation of the medium with nerve growth factor (NGF; 20 ng/ml; Sigma, St. Louis, MO, USA) for 4 days.

### Microwave Exposure and Dosimetry

As described previously [16], the microwave source, a klystron amplifier model JD 2000 (Vacuum Electronics Research Institute, Beijing, China), was capable of generating pulsed microwaves at S-band with the frequency of 2.856 GHz. Microwave energy was transmitted by rectangular waveguide and A16-dB standard-gain horn antenna to an electromagnetic shield chamber. The average power densities were measured using a waveguide antenna, the GX12M1CHP power meter (Guanghua Microelectronics Instruments, Hefei, China) and GX12M30A power heads. The average field power densities were 30 mW/cm^2^.

Rats and PC12 cells were divided randomly into exposure and sham groups. Animals in the exposed group were placed in individual polypropylene cages where they were exposed to microwaves in a temperature controlled room. The temperature of the cell plates was maintained steadily at 37°C with an appropriate heating system. Animals and PC12 cells in the exposed group were exposed to microwaves of a mean power density of 30 mW/cm^2^ for 5 min. To negate any other type of psychophysiological effects, animals and cells in the sham group were processed in parallel to the exposed groups, but without microwave exposure.

The SAR calculation was based on the finite difference time domain (FDTD) method. In our study, the software for calculating SAR of rats was the simulation platform Empire: IMST-Empire v-4.10 (GmbH, Kamp-Lintfort, Germany). The average SAR of whole-body was calculated to be 14 W/kg for the 30 mW/cm^2^ group. The SAR of the cells was determined numerically using the simulated software package Empire V4.10. The medium model with meniscus was based on the profile functions of Schuderer [17]. The PC12 cells in 6-well plates were exposed to 30 mW/cm^2^ at an average SAR of 19 W/kg.

### Measurement of Temperature

The rectal temperatures of rats were measured by thermometer before and after the exposure. The cell supernatant in 6-well plates was measured using an optic fiber thermometer m3300 (Luxtron Corp., Santa Clara, CA, USA) and temperature signals were recorded at a 1.0-Hz sampling rate.

### Morris Water Maze

The water maze task was performed in a circular pool (150 cm in diameter) filled with water maintained at 23±0.5°C in a suitably equipped room at constant temperature, humidity, and brightness. The surface of a clear movable escape platform (12 × 15 cm) was submerged 1.5 cm below the water surface at a specific location for the entire session. The pool was surrounded by thick curtains to hide extra-maze visual cues from the rats.

Rats were trained to find a submerged escape platform, located in a fixed position relative to the extra-maze visual cues, during four consecutive daily sessions. Each session consisted of four trials. Four different starting positions, equally spaced around the perimeter of the pool, were used in a fixed order. Each animal was released into the water and positioned to face the wall of the pool. Each trial had a maximum duration of 60 s and any the rats that did not find the platform within this time were placed on the platform. All rats remained on the platform for 20 s before proceeding to the next trial.

After completion of the four training sessions, 10 rats were exposed to the microwaves, whereas 10 others experienced sham conditions. Assessment of spatial memory was performed at 6 hours, 1 day, 2 days, 3 days, 4 days, 7 days, and 14 days after exposure. The memory test had an identical format to the training session, consisting of four consecutive trials. Rat behavior in the Morris water maze (MWM) experiments during the training and memory test procedures was digitally recorded using a SLY-MW system (Beijing Sunny Instrument Co. Ltd, Beijing, China), and the average escape latency (AEL) was analyzed.

### Protein Extraction and Western Blot

#### Tissues and cells protein extraction

At different time points after microwave exposure, rats were anaesthetized by injecting sodium pentobarbital (80 mg/kg) into the peritoneum with a 27 gauge, half-inch needle. Brain tissues were quickly removed and the hippocampus was excised immediately on the ice. PC12 cells were collected for western blot experiments after neuronal differentiation. Animal and cell samples were homogenized in 400 µl RIPA lysate (Santa Cruz Biotechnology, Santa Cruz, CA, USA) with 1% (v/v) protease inhibitor cocktail (Roche Applied Science, Switzerland) and schizolysised for 30 min on ice. Then the sample was centrifuged at 12000 *g* for 15 min at 4°C and the supernatant was stored at −80°C. A bicinchoninic acid (BCA; Roche Applied Science) protein assay was used for quantification of protein and then the protein was denatured at 100°C for 5 min in 3× sample buffer.

#### Western blot

Proteins (30–50 µg) from each sample were fractionated using 8% sodiumdodecyl sulfate polyacrylamide gel electrophoresis (SDS-PAGE) and transferred onto polyvinylidene fluoride (PVDF) membranes (Merk Millipore, Billerica, MA, USA). The membrane was blocked in 5% low fat milk powder in PBS for 1 hour at 4°C and probed with anti-glyceraldehyde-3-phosphate dehydrogenase (GAPDH; 1∶10000 dilution; Santa Cruz Biotechnology) rabbit polyclonal primary antibody, anti-p-Syn I (ser-62/67, ser-553, and ser-603; 1∶1000 dilution, Santa Cruz Biotechnology), anti-VGAT antibody (1∶1000 dilution; Merck Millipore), anti- VGLUT1 antibody (BNPI; 1∶1000 dilution; Santa Cruz Biotechnology).Bands were visualized by an enhanced chemiluminescence detection system using FluorChem FC2 (Alpha Innotech, USA) and quantified by Photoshop (Adobe software). The intensities of band of interest were expressed relative to the GAPDH intensities from the same sample.

### Immunogold Labeling

#### Animal perfusion and slice preparation

Rats were anesthetized as above, and their brains were fixed by sequential perfusion of heparin in 0.1 M phosphate buffer saline (PBS; pH 7.4) and then 0.05% glutaraldehyde, 15% picric acid, and 4% paraformaldehyde in 0.1 M PBS (pH 7.4), through the left ventricle using a peristaltic pump. After perfusion, the brain was removed from the head and post-fixed in PFA for 2 hours at 4°C. Subsequently, the brain was cut into transverse slices (40 µm) in ice-cold PBS using a vibratome. With a fine brush, sections selected for immunocytochemistry were transferred into a glass vial containing PBS.

#### Pre-embedding immunocytochemistry

First, the sections were removed from the PBS and placed in a fresh solution of 1% sodium borohydride in 0.1 M PBS for 30 minutes at room temperature (RT). They were then incubated for 30 minutes at RT in 1% bovine serum albumin and 0.1% gelatin in PBS at pH 7.4 (PBS-BSA). Next, sections were incubated for 48 hours with the following primary antibodies in blocking solution: rabbit anti-p-Syn I antibody (ser-62/67 and ser-553; 1∶1000 dilution; Santa Cruz Biotechnology), anti-VGLUT1 antibody (BNPI; 1∶1000 dilution; Santa Cruz Biotechnology), and anti-VGAT antibody (1∶1000 dilution; Merck Millipore). Subsequently, the sections were incubated with secondary antibodies (Alexa Fluor488 FluoroNanogold,Nanoprobes, NY, USA) diluted 1∶100 in PBS-BSA with 1% goat serum for 1 hour at RT, and then they were post-fixed with 1% glutaraldehyde in PBS for 10 minutes. Finally, silver enhancement with a HQ silver EM intensification kit (Nanoprobes) was performed for 6 minutes and then the sections were rinsed three times in deionized water for 10 min.

#### Processing for electron microscopy

The sections were immersed in 1% osmium for 30 minutes at RT, then dehydrated using a series of ascending concentrations of ethanol in water, and finally, embedded in resin. A few semi-thin sections (0.5–1 µm) were cut and positioned with the aid of an optical microscope. Silver ultrathin sections were cut (60–80 nm), and stained with uranyl acetate and lead citrate. Examination of these was carried out using a Philips (Amsterdam, Netherlands) CM-120 electron microscope.

### Silencing of Synapsin I via Short Hairpin RNA (shRNA) Transfection

Synapsin I shRNA (GV175) and a scrambled sequence shRNA (control) were constructed using hU6-MCS-CMV-GFP-SV40-Puromycin. The target sequence is located in the C-terminal coding region as 5′ GCAGCTCATCGTGGAACTT 3′ and was synthesized by Genechem Co. (Shanghai, China).PC12 Cells were seeded at 8 × 10^5^/well onto 60 mm dishes for 24 hours prior to plasmid transfection. They were then transfected with a synapsin I shRNA plasmid using Lipofecamine^TM^2000 reagent (Invitrogen) in serum-free Opti-MEM (Invitrogen) for 6 hours. Cells were differentiated by NGF. The transfected cells were grown in complete medium at 37°C and 5% CO_2_. They were harvested at the indicated time points and then used for further analysis.

### Synaptosomes and PC12 Cell Neurotransmitter Release Experiments

#### Preparation of synaptosomes

Unexposed animals were killed, and the hippocampus was quickly removed. Purified synaptosomes were prepared using Percoll gradients as described previously [18], with some minor modifications. The tissue was homogenized in 0.32 M sucrose, buffered at pH 7.4 with Tris. The homogenate was centrifuged (5 min; 1000 *g* at 4°C) to remove nuclei and debris. The supernatant was gently stratified on a discontinuous Percoll gradient (2, 6, 10, and 20% v/v in Tris-buffered sucrose) and then centrifuged at 33.5 × 10^3^ *g* for 5 min. The layer between the 10 and 20% Percoll was collected and washed by centrifugation. When used for neurotransmitter release experiments, synaptosomes were resuspended in physiological medium with the following composition (in mM): 125 NaCl, 3 KCl, 1.2 MgSO_4_, 1.2 CaCl_2_, 1 NaH_2_PO_4_, 22 NaHCO_3_, 10 glucose, pH 7.2–7.4.

#### Neurotransmitter release experiments

Each group of synaptosomes were incubated at 37°C for 30 min with gentle shaking in a 95% O_2_ and 5% CO_2_ atmosphere. Synaptosomes and PC12 cells were stimulated with 1.5 µg/ml KCl prior to microwave exposure. Six hours after exposure, synaptosomes and cells samples were centrifuged at 12000 rpm for 5 min and the levels of aspartate, glycine, glutamate, and GABA in the supernatant (treated with the cell-lysis agent, 10% Sulfosalicylic acid, V/V = 3/2) were determined by a HPLC system.

### Statistical Analysis

Data were represented as the mean ± standard deviation (SD). Comparisons of exposed versus sham groups were analyzed using Student’s *t*-test and multiple group comparisons were analyzed via ANOVA. *P*<0.05 was considered statistically significant.

## Results

### Temperatures Increased Less than 1°C

**Figure 1:**
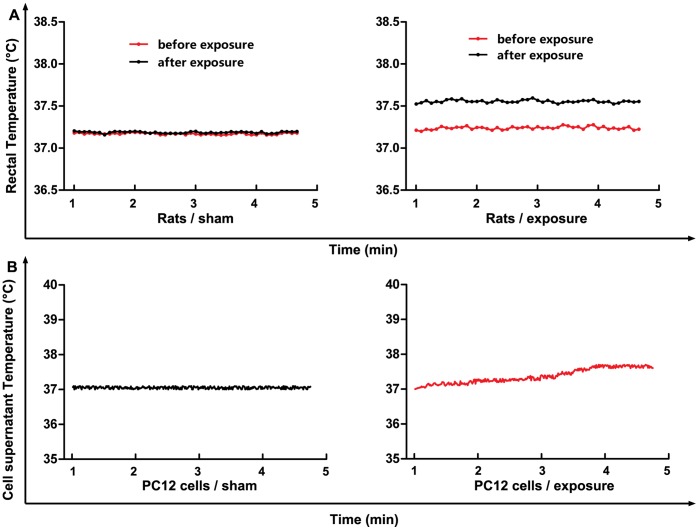
Temperatures increased less than 1°C. **1A and 1B**, Compared to the sham group, the rectal temperatures of rats increased 0.3°C, and the cell supernatant temperatures of PC12 cell line increased 0.7°C in 30 mW/cm^2^ group after 5 min microwave exposure.

We recorded the rectal temperatures and cell supernatant temperatures when rats and PC12 cells exposed by microwave respectively. Compared to the initial temperature, the rectal temperatures of rats increased 0.3°C (Figure 1A), and the cell supernatant temperatures of PC12 cell line increased 0.7°C in 30 mW/cm^2^ group after 5 min microwave exposure (Figure 1B). Therefore, the temperatures in rats and cell supernatant were increased less than 1°C.

### Spatial Memory Ability of Rats Decreased after Microwave Exposure

**Figure 2:**
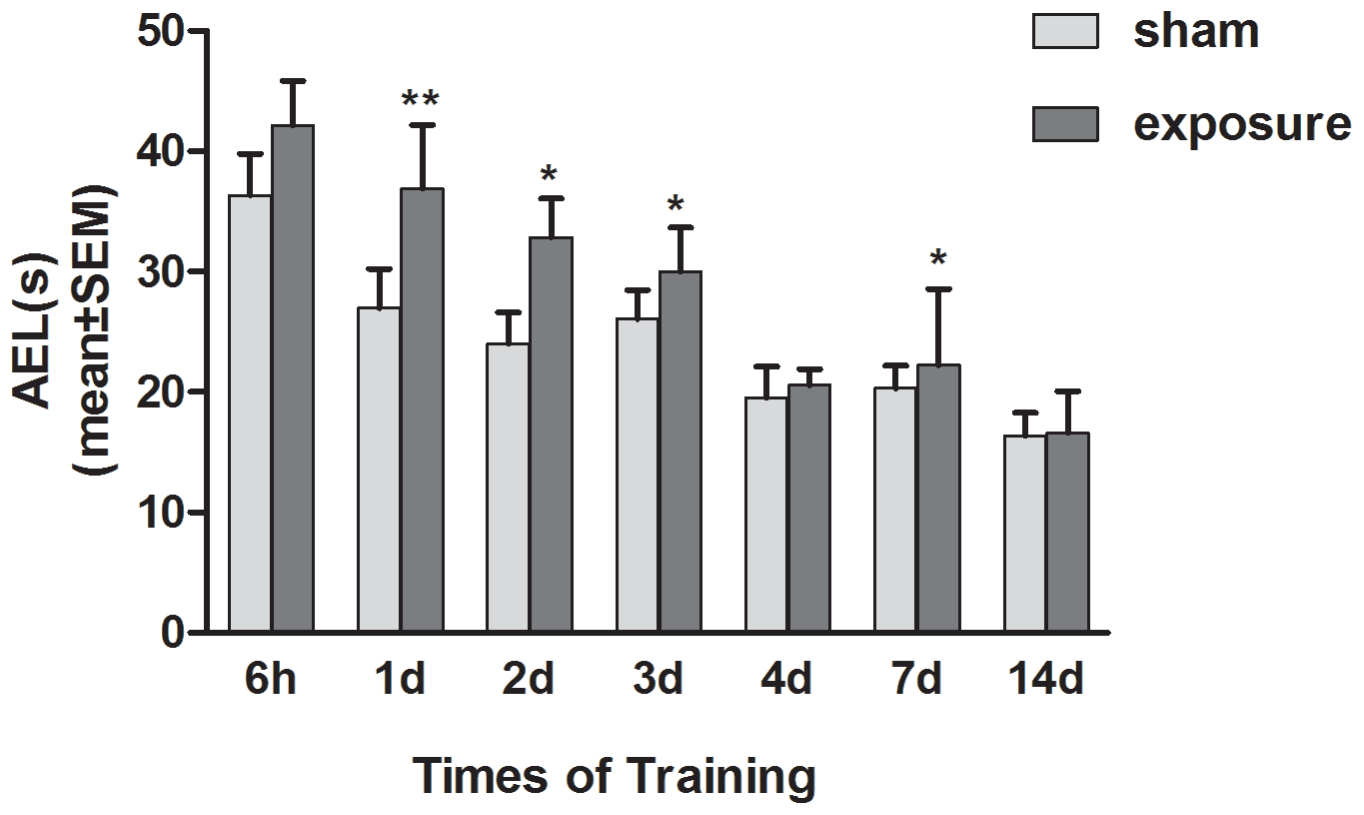
Spatial memory performance of Wistar rats decreased after microwave exposure. Compared to the sham group, the Average escape latency (AEL) of rats in Morris water maze navigation task were significantly longer in the exposure group at 1 day (P<0.01), 2 day (P<0.05), 3 day (P<0.05) and 7 day (P<0.05) after exposure. Data represent mean ± SD. *P<0.05 versus sham group. **P<0.01 versus sham group.

We tested the spatial memory performance of Wistar rats exposed to microwaves for 5 min, using the Morris water maze (MWM). As shown in Figure 2, compared to the sham group, the AEL of rats in the exposed group were significantly longer at day 1, 2, 3, and 7 after exposure (*P*<0.05). Animals exposed to the microwaves were spending more time to retrieve the location of the submerged platform that was learned during the training period. By contrast, the sham-exposed rats exhibited a clear preference for the quadrant in which the platform was located during training, showing that they had consolidated the learned information and could effectively retrieve it.

### Attenuated GABA Release in Wistar Rats after Microwave Exposure

**Figure 3:**
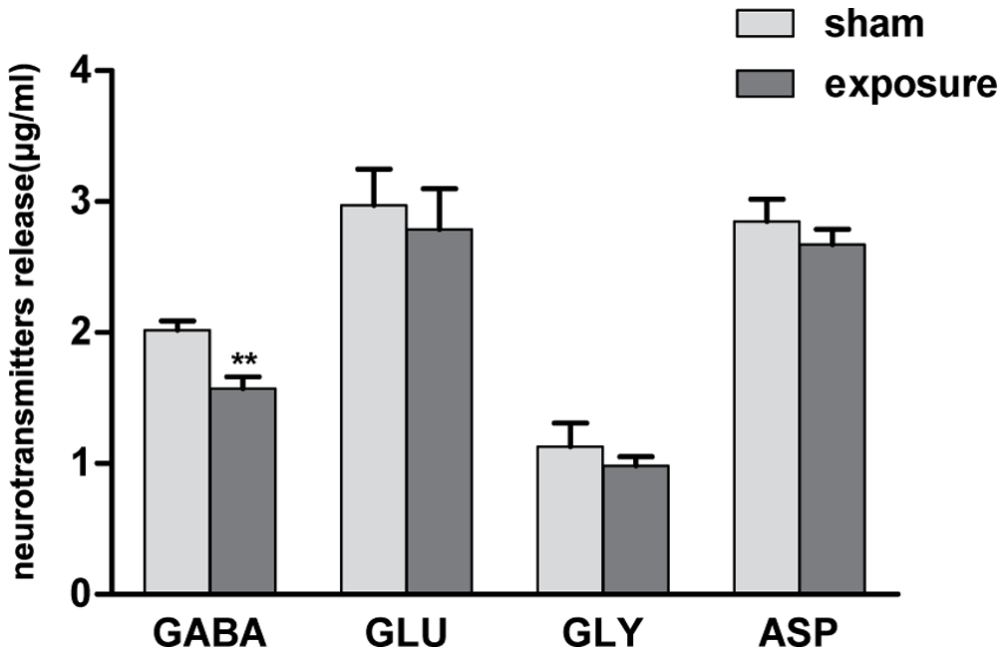
Attenuated release of GABA in Wistar rats after microwave exposure. The release of GABA was attenuated significantly compared to the sham rats (P<0.01). Data represent mean ± SD. **P<0.01 versus sham group.

Synaptosomes were purified from the hippocampal tissue of both sham and exposed rats. Amino acid neurotransmitter (GABA, glutamate, glycine, and aspartate) release from the synaptosomes was determined by HPLC at 6 hours after exposure. As shown in Figure 3, the release of GABA was significantly decreased in the microwave exposed group versus the sham group (*P*<0.01).

### Protein Expression Diversity of p-Syn I, VGAT and VGLUT in Wistar Rats after Microwave Exposure

**Figure 4:**
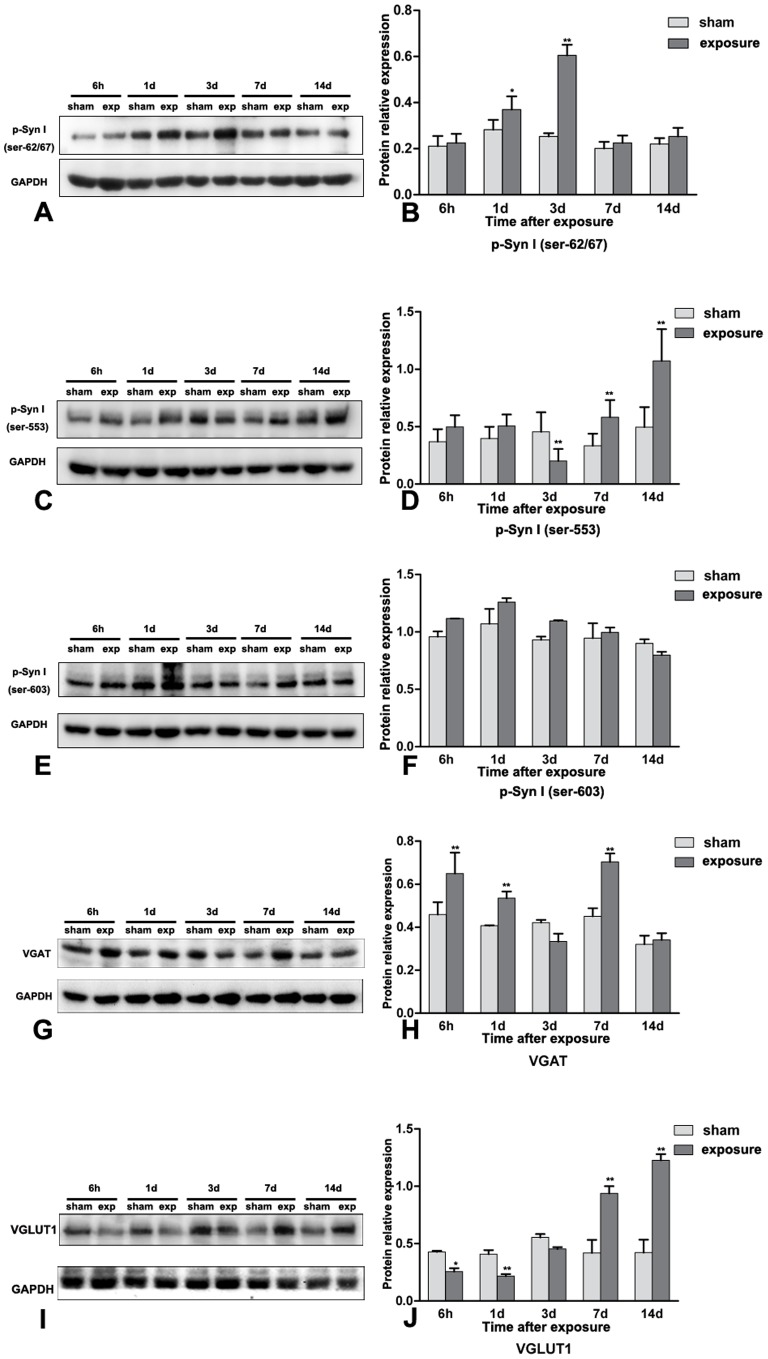
Protein expression diversity of p-Syn I, VGAT and VGLUT in Wistar rats after microwave exposure. **4A and 4B**, p-Syn I(ser-62/67) protein expression was increased significantly at 1 day and 3 day after the exposure (P<0.05 and P<0.01, respectively); **4C and 4D**, the expression of p-Syn I (ser-553) was decreased at 3 day after the exposure (P<0.01) with subsequent elevated protein expression at 7 day and 14 day (both P<0.01). **4E and 4F**, no protein expression variation was observed for p-Syn I (ser-603). **4G and 4F**, VGAT protein expression was significantly elevated at 6 hour, 1 day and 7 day after exposure. **4I and 4J**, VGLUT1 was found down-regulated at the beginning (6 hour and 1 day) with significant up-regulation at 7 day and 14 day. Data represent mean ± SD. *P<0.05 versus sham group. **P<0.01 versus sham group.

As shown in Figures 4A and 4B, p-Syn I (ser-62/67) protein expression was increased significantly at days 1 and 3 in exposed rats (*P*<0.05 and *P*<0.01, respectively), whereas there was no difference at day 7. In contrast, exposure correlated with decreased expression of p-Syn I (ser-553) at day 3 (*P*<0.01) and increased expression at days 7 and 14 (Figure 4C and 4D; both *P*<0.01). No differences in protein expression were observed for p-Syn I (ser-603), as shown in Figures 4E and 4F. Vesicular GABA transporter (VGAT) protein expression (Figures 4G and 4H) was significantly elevated in the exposed group at 6 hours, 1 day and 7 days (*P*<0.05). Vesicular glutamate transporter 1 (VGLUT1; Figures 4I and 4J) was found to be down-regulated at early time points (6 hour and 1 day; *P*<0.05) whereas significant up-regulation was observed in later measurements.

### Alterant Distribution of p-Syn I (ser-553) and VGAT in Wistar Rats after Microwave Exposure

**Figure 5:**
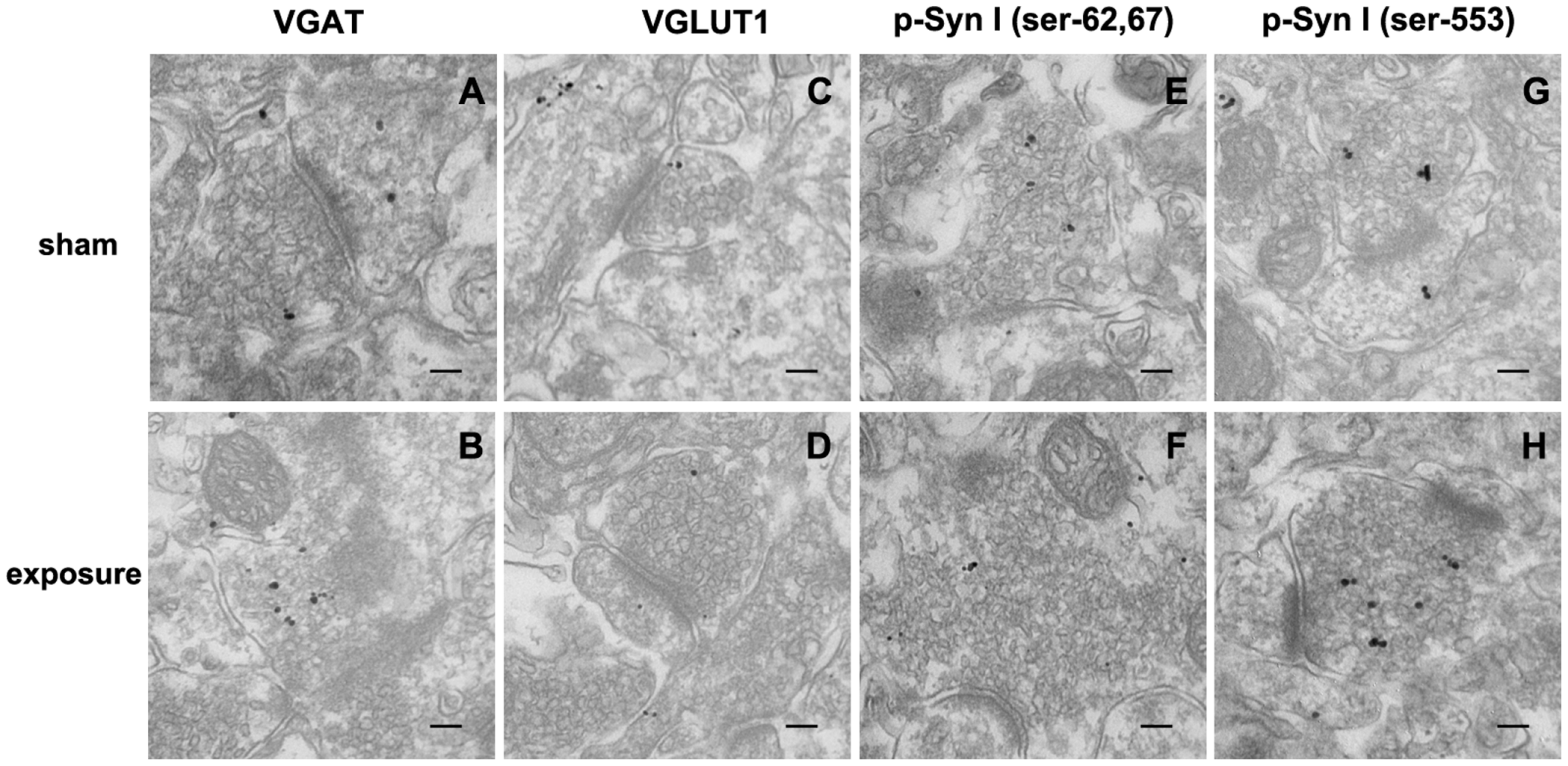
Alterant distribution of p-Syn I (ser-553) and VGAT in Wistar rats after microwave exposure. 5B, 5D, 5F and 5H, numerous small clear synaptic vesicles gathered in presynaptic terminal. Meanwhile, the immunogold particles distribution of p-Syn I (ser-553) (5H) and VGAT (5B, dark spots) were both enriched in these vesicles while VGLUT1 and p-Syn I (ser-62/67) remained unchanged. (Scale bar, 100 nm).

In order to determine the synaptic distribution and subcellular location of p-Syn I, VGAT and vesicular glutamate transporter 1(VGLUT1) proteins, we observed the distribution profiles of these proteins in the hippocampal CA3 region by pre-embedding immune electron microscopy. As illustrated in Figure 5, at 7 days after microwave exposure, numerous small clear synaptic vesicles gathered abnormally in presynaptic terminals. Additionally, the immunogold labeling of p-Syn I (ser-553) and VGAT was increased in these vesicles while VGLUT1 and p-Syn I (ser-62/67) remained unchanged.

### Protein Expression Diversity of p-Syn I (ser-553) in PC12 Cell Line after Microwave Exposure

**Figure 6:**
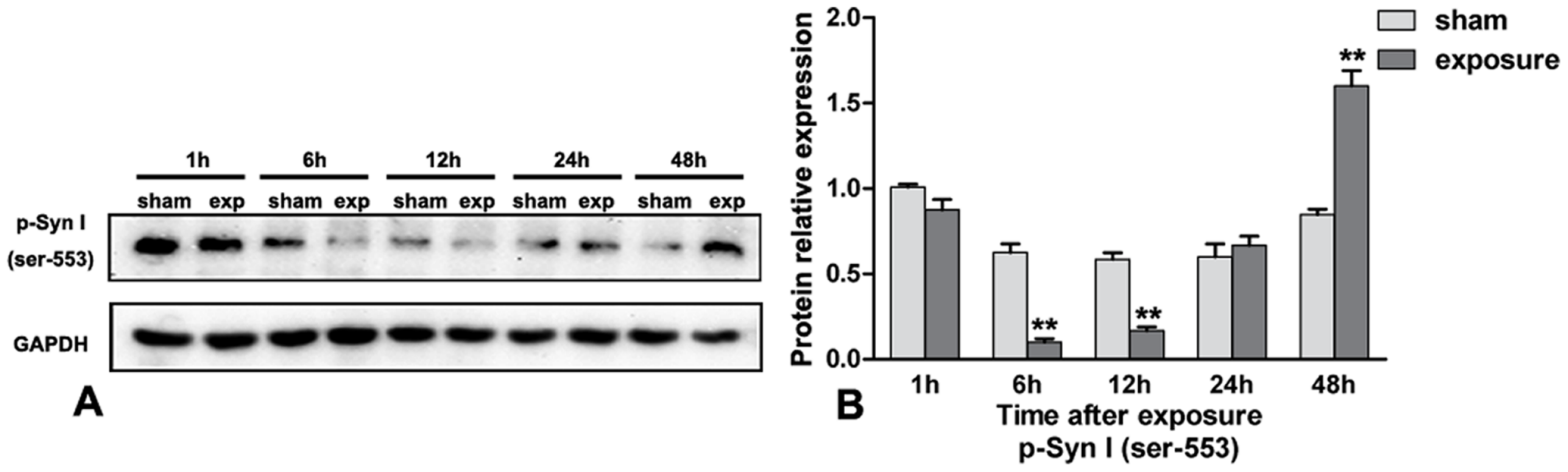
Protein expression diversity of p-Syn I (ser-553) in PC12 cell line after microwave exposure. **6A–6B**, the expression of p-Syn I(ser-553) was attenuated at 6 hour and 12 hour after exposure (P<0.01) with subsequent elevated protein expression at 48 hour (P<0.01).Data represent mean ± SD. *P<0.05 versus sham group. **P<0.01 versus sham group.

After protein extraction, p-Syn I expression was detected by western-blot assay. As shown in Figures 6A and 6B, the protein expression of p-Syn I (ser-553) was greatly attenuated at 6 hours and 12 hours after the 5 min microwave exposure (both *P*<0.01) with subsequent elevated protein expression 48 hour.

### Silencing p-Syn I Resulted in the Reduction of GABA Releasing

We next intended to explore the relationship between p-Syn I expression alteration and GABA releasing reduction. First, PC12 cell line was transfected with synapsin I shRNA to silence its expression. Based on microwave exposure and transfection with synapsin I-silencing shRNA, PC12 cells were divided at random into four groups: 1) sham exposure with control shRNA transfection (sham+control), 2) microwave exposure with control shRNA transfection (expose+control), 3) sham exposure with synapsin I shRNA transfection (sham+GV175), and 4) microwave exposure with synapsin I shRNA transfection (expose+GV175). Finally, p-Syn I expression and neurotransmitter release were determined by western blot assay and HPLC, respectively.

**Figure 7:**
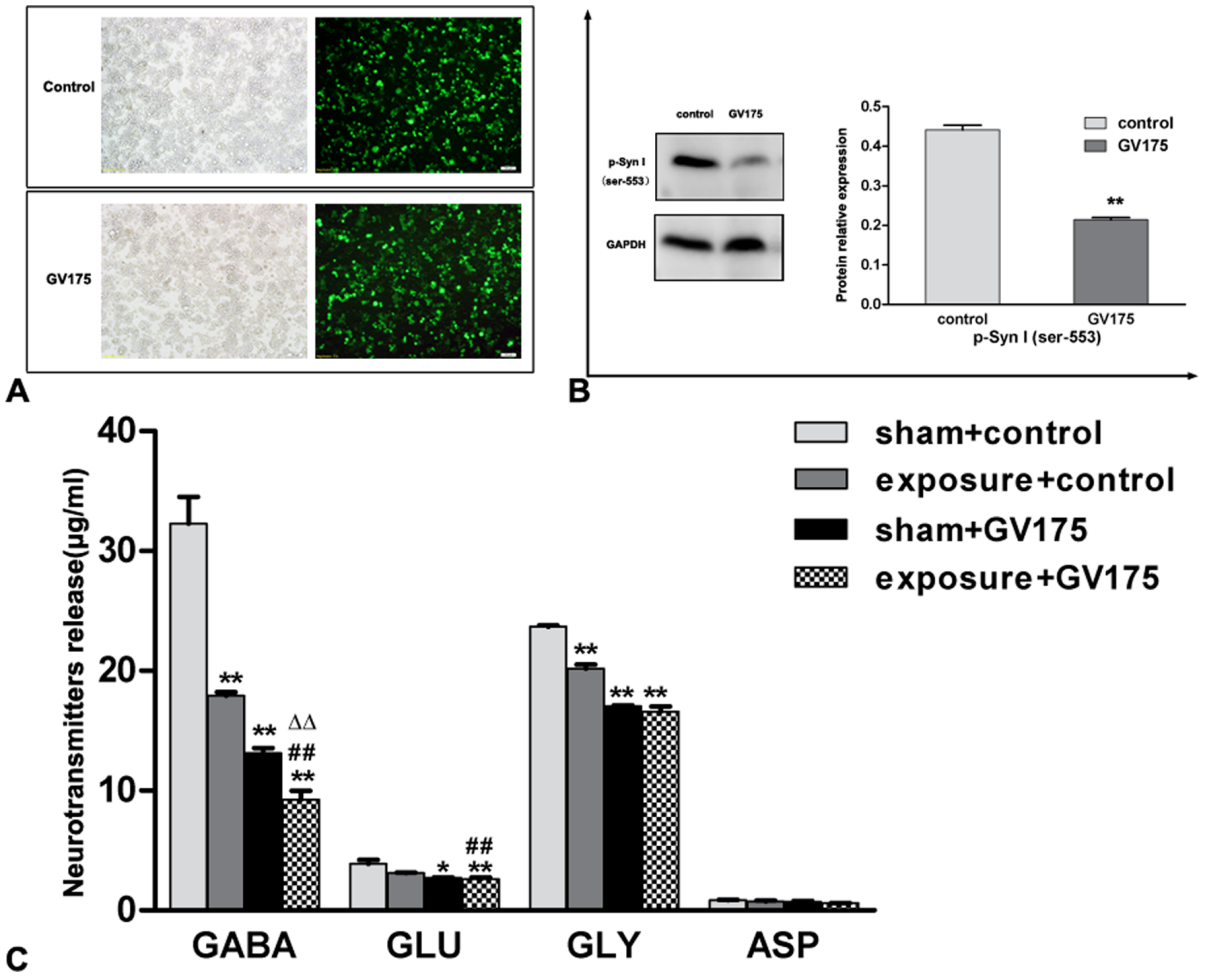
P-Syn I silencing resulted in the reduction of GABA release. (A) Bright field and fluorescence micrographs demonstrating transfection of synapsin I shRNA (green). (B) Protein expression of p-Syn I (ser553) was decreased after silencing of the Synapsin I gene. (C) The release of GABA, glutamate (GLU), glycine (GLY), and aspartate (ASP) was measured by HPLC at 6 hours after microwave exposure, with and without silencing (GV175) and control, respectively. The release of GABA and other neurotransmitters were significantly attenuated after microwave exposure and p-Syn I silencing. Data represent mean ± SD. *P<0.05 versus sham+control group, ** P<0.01 versus sham+control group; ^##^ P<0.01 versus expose+control group. ^ΔΔ^ P<0.01 versus sham+GV175 group.

As shown in Figures 7A and 7B, p-Syn I (ser-553) were found to be significantly down-regulated after shRNA transfection (*P*<0.01). In the sham+GV175 group, GABA release was decreased after silencing of Synapsin I (Figure 7C). Similar to the previous results on rats (Figure 3), GABA release was observed to be diminished in the expose+control group after microwave exposure. Maximal reduction of GABA release was found for the expose+GV175 group. Glutamate and glycine levels were also found to be diminished as a result of microwave exposure and/or p-Syn I silencing.

## Discussion

In this study, we set out to investigate the potential role of phosphorylated synapsin I in the microwave-induced impairment of synaptic transmission and spatial memory; thereby shedding light on the health risks posed by microwave exposure. In this study, the recorded temperature of rats and cell culture fluid during exposure slightly increased for less than 1°C in the 30 mW/cm^2^, indicating that the non-thermal effects are dominant.

Changes in behavior and cognition are important outcomes for assessment of the effects of microwave exposure on the brain [19]. In the present study, the hippocampus-dependent spatial learning and memory of rats exposed to microwaves was examined using the Morris water maze navigation task. The time taken to complete the task after training (AEL) was increased in the microwave-exposed group, which suggested that microwave exposure disrupted spatial learning and memory in the rats [20]–[22].

Our previous studies found that the hippocampus can be injured by microwave exposure, resulting in the impairment of cognitive function, which may be partly due to attenuation of neurotransmitter release and abnormal expression of synaptic vesicular proteins [23], [24]. The present study has confirmed that such attenuation of neurotransmitter release does occur, showing that synaptosomal GABA release was reduced by microwave exposure. It has been reported that the MWM task causes a lasting increase in GABA release [25]. Additionally, various study show that unbalanced GABA release triggered cognition deficits in rat and intellectual disability in human [26], [27]. This indicates that GABAergic transmission plays a role in spatial learning and memory, and that our neurotransmitter release results are consistent with our MWM results.

Numerous studies have shown that synapsin I phosphorylation at serine 553 and 62/67 sites increases the probability of neurotransmitter release from synaptic vesicles [28], [29]. However, the potential relationship of p-Syn I to the aberrant neurotransmitter release associated with microwave-induced impairment of learning and memory has been poorly understood. Elucidating this mechanism was the primary concern of this study.

To investigate whether p-Syn I along with GABA and glutamate transporters were involved in attenuated GABA release, we measured their expression. The results indicated that expression of p-Syn I (ser-553) was decreased at day 3 after microwave exposure whereas it was elevated at later time points. VGAT was also significantly elevated in hippocampal tissue post-exposure. This implies that p-Syn I (ser-553) may be involved in the abnormal release of GABA that is induced by microwave radiation. We then observed the presynaptic distribution of these proteins at day 7 after exposure and found that both were enriched in small clear presynaptic vesicles. These data provide further support for the role of p-Syn I (ser-553) in the attenuated release of GABA after microwave exposure, which may occur in part through altered expression and distribution of p-Syn I (ser-553), which could be identified as a critical characteristic of behavioral and cognitive dysfunction by attenuating GABA release during synaptic vesicle exocytosis in the hippocampus of microwave exposure rats.

On the other hand, the distribution of VGLUT1 and p-Syn I (ser-62/67) remained unchanged at day 7 after exposure, and glutamate release along with p-Syn I (ser-603) expression showed no obvious changes after irradiation. Taken together, we must conclude that attenuated GABA release after exposure is not associated with changes in p-Syn I (ser-62/67) or p-Syn I (ser-603).

The impairment in GABA transmission has been previously observed in synapsin I knockout mice [30], [31] and myriad studies have found that mutation and deletion of the synapsin I gene results inimpairment of memory and synaptic plasticity in humans and animals by triggering imbalances in the dynamics of GABA inhibition [27], [32], [33]. In the present study, we used a gene knockdown experiment to further explore the relationship between p-Syn I (ser-553) expression and GABA release after microwave exposure of PC12 cells. We observed that the protein expression of p-Syn I (ser-553) and the release of GABA were both decreased at 6 hours after exposure and that GABA release was reduced when expression of p-Syn I (ser-553) was silenced. These results show that microwave exposure and knockdown of p-Syn I (ser-553) have very similar outcomes and that p-Syn I (ser-553) is essential for inhibitory transmission, which was consistent with some other reports [34]. Our results also showed that the release of GABA was further reduced by the combination p-Syn I (ser-553) silencing and microwave exposure, indicating a synergetic effect. Finally, p-Syn I silencing also reduced glutamate and glycine levels, which is consistent with the important parts they play in learning and memory.

Although beyond the scope of this study, p-Syn I is involved in multiple stages of vesicle cycling, including vesicle mobilizing, docking, priming, and fusion [35], [36]. Future studies should address the effect of microwaves on these specific stages of the GABA release mechanism and further elucidate the role of p-Syn I in this process. Such detailed mechanistic information, combined with that already revealed by the present study, may facilitate new approaches to the treatment and prevention of microwave-related brain disease.

## Conclusions

Our experiments on Wistar rats and PC12 cell line suggest that microwave exposure (30 mW/cm^2^) can induce behavioral and cognitive dysfunction via altered GABAergic transmission. We have shown for the first time that reduced p-Syn I (ser-553) expression plays a critical role in this effect.
